# Recent Advances of Hierarchical and Sequential Growth of Macromolecular Organic Structures on Surface

**DOI:** 10.3390/ma12040662

**Published:** 2019-02-22

**Authors:** Corentin Pigot, Frédéric Dumur

**Affiliations:** Aix Marseille Univ, CNRS, ICR UMR 7273, F-13397 Marseille, France

**Keywords:** on-surface reaction, stepwise growth, sequential growth, hierarchical growth, macromolecular organic structures, surface covalent organic framework, nanoribbons, macrocycles, coordination polymers

## Abstract

The fabrication of macromolecular organic structures on surfaces is one major concern in materials science. Nanoribbons, linear polymers, and porous nanostructures have gained a lot of interest due to their possible applications ranging from nanotemplates, catalysis, optoelectronics, sensors, or data storage. During decades, supramolecular chemistry has constituted an unavoidable approach for the design of well-organized structures on surfaces displaying a long-range order. Following these initial works, an important milestone has been established with the formation of covalent bonds between molecules. Resulting from this unprecedented approach, various nanostructures of improved thermal and chemical stability compared to those obtained by supramolecular chemistry and displaying unique and unprecedented properties have been developed. However, a major challenge exists: the growth control is very delicate and a thorough understanding of the complex mechanisms governing the on-surface chemistry is still needed. Recently, a new approach consisting in elaborating macromolecular structures by combining consecutive steps has been identified as a promising strategy to elaborate organic structures on surface. By designing precursors with a preprogrammed sequence of reactivity, a hierarchical or a sequential growth of 1D and 2D structures can be realized. In this review, the different reaction combinations used for the design of 1D and 2D structures are reported. To date, eight different sequences of reactions have been examined since 2008, evidencing the intense research activity existing in this field.

## 1. Introduction

**Figure 1:**
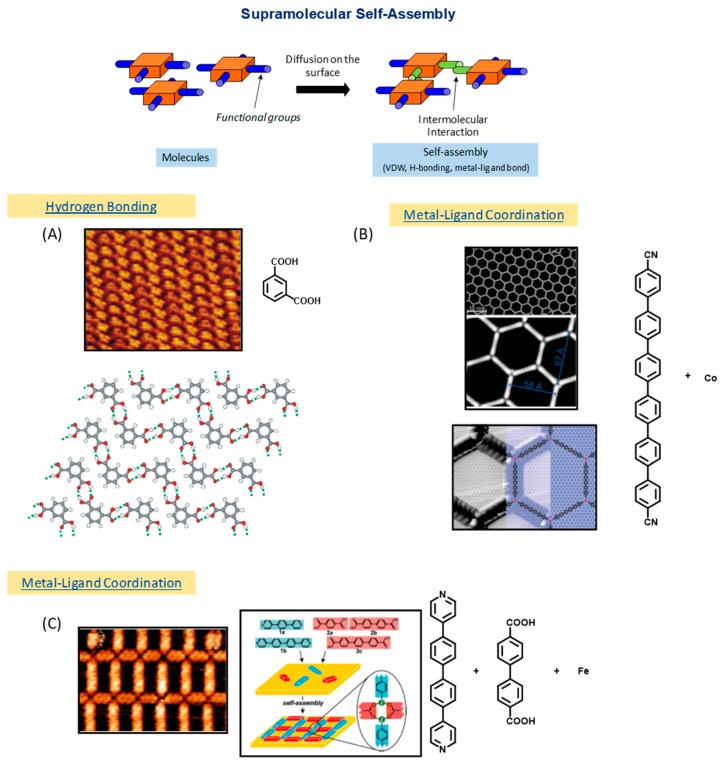
Principle of the supramolecular self-assembly. (**A**) Scanning tunneling microscope (STM) image of isophthalic acid on highly ordered pyrolytic graphite (HOPG) and picture of the molecular mechanics simulation. Adapted with permission from Lackinger et al. [3]. Copyright 2004 American Chemical Society. (**B**) STM images of the metal–organic supramolecular structure formed with cobalt on Ag(111) and superimposition of a ball and stick model onto the STM image. Reprinted with permission from Kühne et al. [5]. Copyright 2009 American Chemical Society. (**C**) STM image of the iron coordination network and schematic illustration of the complementary ligands coordinated to iron, Langner et al. [6]. Copyright 2007 National Academy of Sciences.

The discovery of new classes of materials is fundamental to the development of future applications [1] and the possibility to elaborate well-organized structures on surface paves the way towards the design of miniaturized devices and the exploration of new concepts in nanoelectronics [2]. With regards to their exquisite properties, organic compounds are prime candidates because their functionalities are widely variable. During decades, supramolecular chemistry has proved to be a powerful tool to elaborate well-organized and extended structures on surfaces [3,4,5,6] (see Figure 1). By applying the fundaments of supramolecular chemistry, the monomers which constitute the elemental building-blocks for the construction of two-dimensional structures could be arranged precisely, with a long-range order by mean of secondary interactions (van der Waals interactions, hydrogen bonds, etc.). 

**Figure 2:**
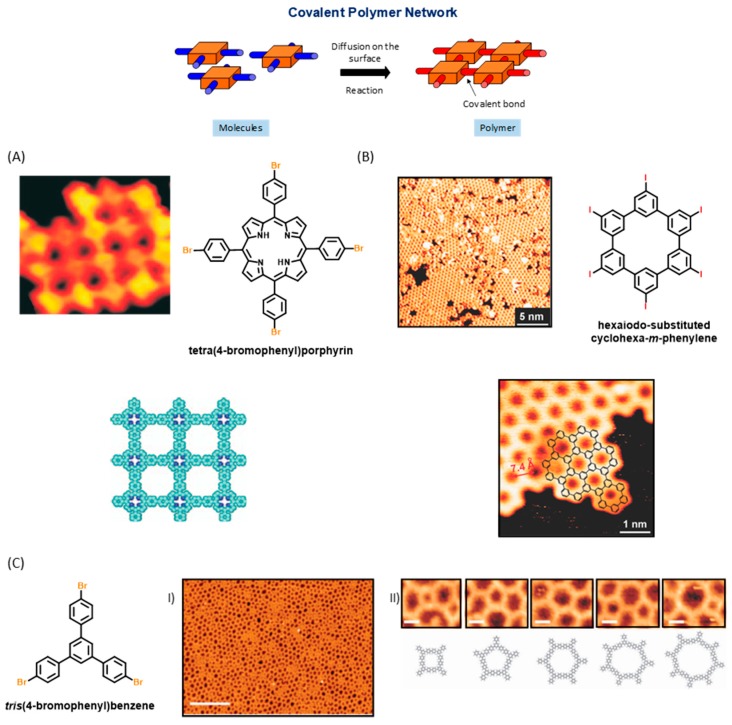
Principles of the formation of covalent polymer network on surface. (**A**) STM image of the polymerization of tetra(4-bromophenyl)porphyrin on Au(111). Reprinted by permission from Springer Customer Service Center GmbH: Springer Nature, Nature Nanotechnology, Grill et al. [18], 2007. (**B**) STM image of the polymer formed with hexaiodo-substituted cyclohexa-*m*-phenylene on Ag(111). Reproduced from [19] with permission from The Royal Society of Chemistry. (**3**) STM image of the polymer formed with *tris*(4-bromophenyl)benzene on Au(111). Scale bars: (I) 190 Å, (**II**) 14 Å. Adapted from [20] with permission from The Royal Society of Chemistry

Parallel to this, due to the noncovalent character of the bonds created between tectons [7,8,9,10], defects formed during the initial growth of the supramolecular phase can be partially or totally repaired, self-healing consisting in a thermal post-treatment of the surface. Resulting from the possibility to suppress defects, networks perfectly ordered and extending over tens of square nanometers could be prepared (see Figure 1). By chemical engineering of the molecular tectons, the design of complex structures with precise shapes, compositions and functionalities is thus rendered possible. The large number of published studies and review articles dealing with supramolecular self-assemblies at surfaces demonstrates it as a mature field. However, the low thermal stability and the lack of electronic conjugation often evidenced in these assemblies drastically limit the applicability of these systems [11,12,13]. To overcome these drawbacks, an efficient strategy consists in generating covalent bonds between tectons, but it requires the monomers to be conveniently functionalized (see Figure 2) [14,15,16,17,18,19,20]. 

In this field, formation of covalent bonds between tectons is not limited to two-dimensional (2D) structures and one-dimensional (1D) structures have also been the purpose of numerous studies. Due to the remarkable robustness resulting from the formation of covalent bonds between molecular subunits, the excellent thermal, mechanical and chemical stability, and the possibility to create fully conjugated structures with the desired functionalities, a great impact of these covalent structures in nanoelectronics can already be anticipated, irrespective of their shapes, sizes, and lengths [21]. As far as 2D polymers are concerned, the most basic organic material with a strictly 2D character is graphene, which is composed solely of carbon atoms, and which is attracting considerable interest nowadays as illustrated, in particular, by year 2010’s Nobel Prize award in Physics. Under intense investigations by various groups in the world, the properties of graphene appear simply exceptional in terms of basic science as well as for practical applications in electronic devices [22]. However, applications such as solar cells [23] or field-effect transistors [24] require a high level of control on its doping level to pertain its properties in devices, for example by introducing chemical changes [25]. Indeed, graphene’s properties are very sensitive to chemical modifications [26], which can in principle be extended infinitely to form a well-ordered sheet composed of various heteroatoms. The development of 2D polymers is thus a meeting point where the extraordinary properties of a 2D material such as graphene can be combined with the powerful knowledge of supramolecular and synthesis chemistry to create a new class of materials of quasi-infinite structural and functional diversity [27,28,29]. However, growth control of these structures remains very delicate and there is still a lot of work to be done to improve to optimize the on-surface synthesis.

**Figure 3:**
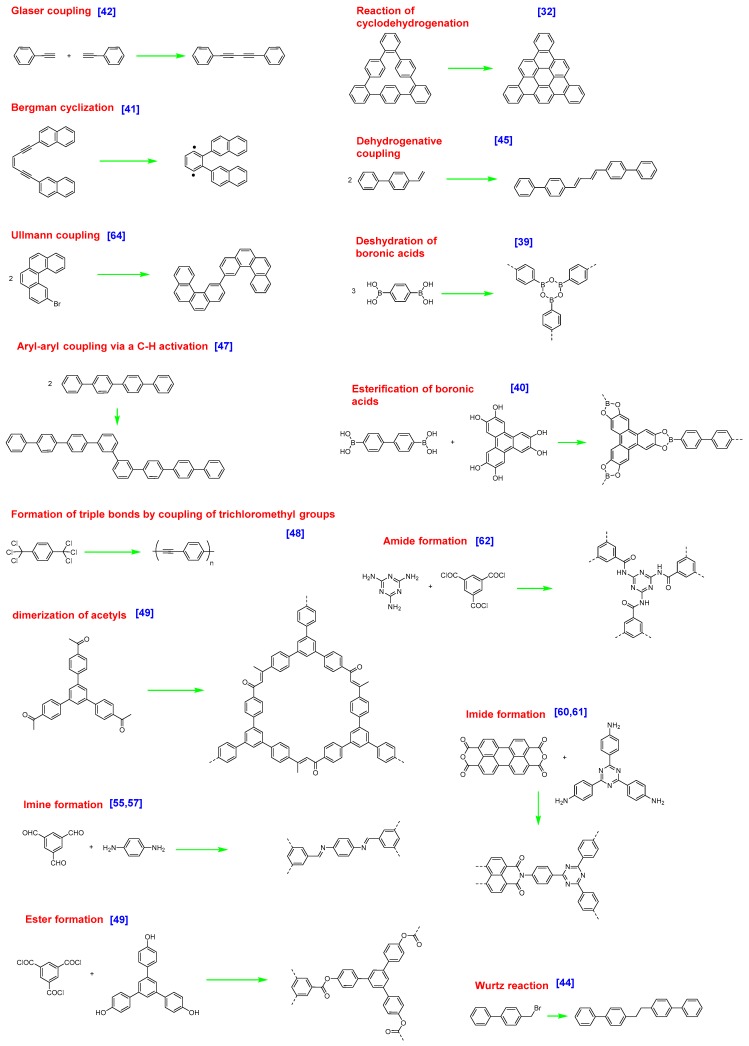
Examples of common on-surface reactions reported in the literature.

At present, possibilities offered by organic chemistry to form covalent bonds between molecular tectons have only been scarcely explored by physicists with regards to the number of existing coupling modes [30,31] and the reactions of cyclodehydrogenation [32,33], dehydration of boronic acids [34,35,36,37,38,39], esterification of boronic acids [40], Bergman cyclization [41], Glaser coupling [42,43], Wurtz reaction [44], dehydrogenative coupling of terminal alkenes [45], dehydrogenative coupling of porphyrines [46], aryl–aryl coupling via a C–H activation [47], formation of triple bonds by coupling of trichloromethyl groups [48], cyclotrimerization of acetyls [49], formation of imines [50,51,52,53,54,55,56,57,58], esters [59], imides [60,61], amides [62,63], or the Ullmann coupling between halogenated aromatic rings [18,64] can be cited as examples of reactions already examined on surface (see Figure 3). Parallel to known reactions, unprecedented coupling modes were also discovered in the context of the surface-mediated reactions, as exemplified with the oxidative coupling of activated methylene groups [65,66,67], the polymerization of alkanes [68], or the aryl–aryl coupling of none halogenated polyaromatics [47] or porphyrins [69], reactions that have never been reported in solution phase chemistry [70].

**Figure 4:**
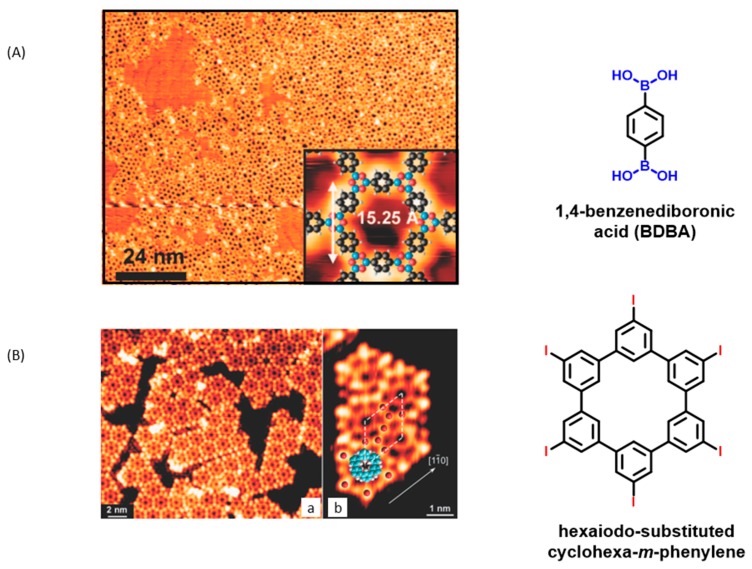
(**A**) Disorder exists during the polymer growth of 1,4-benzenediboronic acid (BDBA) on Au(111). Reprinted with permission from Zwaneveld et al. [39]. Copyright 2008 American Chemical Society. (**B**) Loss of iodine atoms during the thermal deposition of hexaiodo-substituted cyclohexa-*m*-phenylene on a Ag(111) surface kept at room temperature (red spheres correspond to iodine atoms). (**Ba**) STM image of the macromolecular structure on Ag(111); (**Bb**) STM image, magnification of a nanodomain. Adapted from [19] with permission from The Royal Society of Chemistry

In the search for nanostructures of high thermal, mechanical, and chemical stability, a major breakthrough has thus been achieved with the demonstration that the formation of covalent bonds between tectons was possible [18,19,20]. However, rapidly, pioneering works on surface covalent organic frameworks (sCOFs) have evidenced the difficulties of the polymer growth. Notably, due to the formation of covalent bonds and the low-dimensional environment of the on-surface syntheses, possibilities of defect self-healing remain limited, and can even be considered as being precluded for certain reactions such as Ullmann coupling [64], Wurtz coupling [45], or Bergman cyclization [41]. In these conditions, the resulting macromolecular organic structures formed with these reactions are either poorly ordered and limited in terms of 1D or 2D growth. Conversely, all reactions based on a dehydration process are reversible and the possibility of self-healing remains possible, as exemplified with imines [55,57] or boronic esters [40]. To increase the size and the regularity of the nanodomains made of 1D or 2D structures, novel and more efficient chemistries or synthetic approaches offering a better control of the growth conditions are highly desired and will guide the future development of this emerging technology. Concerning growth control, the best candidates to examine this point are undoubtedly 2D structures requiring the covalent linkage to be carried out in the two dimensions and on the basis of the different works reported in the literature, some conclusions can be deduded. Notably, the simultaneous presence of numerous identical and reactive functional groups onto molecular tectons was identified as favoring the formation of numerous defects, the probability for the monomer to react in an inappropriate orientation being drastically increased [71]. This drawback was notably evidenced with diboronic acids during the formation of 2D polymers. Due to an insufficient space to promote the diffusion and the rearrangement of 1,4-benzenediboronic acid (BDBA) onto the surface, various pore shapes could be found on the surface while using this molecular building block (see Figure 4) [29].

Another relevant example of a drawback induced by the presence of numerous reactive functional groups onto the same monomer is provided with hexaiodo-substituted cyclohexa-*m*-phenylene [19]. In this last case, during the thermal deposition of the monomer onto a Ag(111) surface, an undesired dehalogenation reaction occurred, promoting the formation of numerous defects on the surface (see Figure 4). 

To circumvent these different problems, alternative procedures to construct nanoporous structures have been actively researched. In most of the works reported in the literature, molecular tectons only possess a single type of functional groups, inducing a disorder during polymerization as several functional groups of a same monomer are simultaneously involved in reactions. Loss of functional groups is also occasionally observed.

Recently, the development of monomers bearing different types of functional groups activable in different reaction conditions has emerged as an effective way to address the defect issue. Typically, monomers developed for this purpose possess two (or more) functional groups that can be activated separately. More precisely, the dormant sites can be activated in a predetermined order, giving rise to a sequential growth of the final network. The reactive sites are also selected to be compatible so that no reaction should occur between the different functional groups. Such reactions that do not interfere each other are typically named orthogonal reactions [72,73,74,75,76]. Parallel to the sequential growth of polymers, the hierarchical growth of sCOF can also be employed but requires the monomers to be substituted adequately. As the main difference between the sequential and the hierarchical growths of macromolecular architectures, two different types of functional groups are involved contrarily to the hierarchical growth for which the same types of functional groups can be used, but can be activated in different reaction conditions. In this field, the most representative example concerns aromatic precursors bearing two different types of halogens, namely bromines and iodines. Even if an Ullmann coupling can occur with the two types of halogens, selectivity will arise from the difference of reactivity between iodines and bromines. By perfectly controlling the reaction temperature, Ullmann coupling will occur for the iodinated sites prior to the brominated ones. Using this stepwise strategy (hierarchical or sequential), nanoporous architectures extending over tens of square nanometers can be obtained, resulting from an advanced polymerization process at each step. During the last decade, major breakthroughs have been achieved in the hierarchical and sequential growth of 2D polymers. However, this synthetic approach is not limited to 2D structures and 1D structures such as ribbons, macrocycles, and linear polymers have also been prepared using this innovative strategy. Indeed, the contribution of 1D and 2D structures in the comprehension of the underlying mechanisms of polymerization are comparable, especially due to the fact the reactions involved in the sequential or hierarchical growth of 1D and 2D organic structures are different at only one exception. Indeed, a recent work succeeded to combine the Ullmann/aromatization sequential growth strategy typically used for the elaboration of 1D structures to the deshydrogenative cross-coupling of aromatics, enabling the bottom-up synthesis of two dimensional graphene-like structures [77]. To image these structures and the different growth steps of the macromolecular organic structures, scanning tunneling microscopy (STM) is a remarkable technique combining the atomic resolution with the ability to probe the local electronic structure [78]. To get a deeper insight into the chemical structures of the macromolecular organic structures, STM can be combined with other surface analysis methods such as Raman spectroscopy, photoemission, high resolution electron energy loss spectroscopy (HREELS), or synchrotron radiation analyses [79,80]. The combination of these different techniques is of crucial importance, especially to characterize the multistep growth of macromolecular organic structures. Before continuing, a distinction should be made between “self-assembly”, which is commonly used to evoke the formation of both supramolecular and covalent phases on-surface, and “chemically released diffusion”. Indeed, numerous published or being published articles comprise the terminology "self-assembly” whereas the right term is “chemically released diffusion”. Indeed, “self-assembly” refers to the self-organization of molecular tectons on surface without necessarily involving a chemical modification of the molecular tectons and this terminology is well-adapted for supramolecular phases for which the cohesion is ensured by weak intermolecular interactions between elemental building blocks. Contrastingly, chemically released diffusion refers to mass transfer occurring in polycondensates due to the permanent exchange of fragments existing between molecular segments under growth (i.e., via transreactions). This concept developed in the early 1980s by Prof. Stoyko Fakirov is more adapted to describe the formation of covalent phases on surface, the chemical composition of the polymer under growth continuously evolving by exchange reactions [81].

In this review, an overview of the recent advances concerning the sequential or hierarchical growth of macromolecular organic structures on surface in ultrahigh vacuum (UHV) environment is presented. As compared to their one-step counterparts, multistep formation of organic structures on surface is less documented in the literature. However, elaboration of covalent bonds in multistep procedures offers a unique opportunity to guide the growth of the macromolecular architecture and to control the kinetic of reaction at each individual step. This hierarchical and sequential approach is of prime importance for the regularity of 2D polymers on surface, but also for the preparation of 1D structures as the efficacy of the covalent couplings will determine the length of the final structures. At present, most of the reaction combinations used for the sequential growth of 1D and 2D structures are different. To date, eight different combinations of reactions have been examined in the literature and these strategies are presented in this review. 

## 2. Coupling Modes Used for the Design of 2D Covalent Networks

### 2.1. Hierarchical Growth of Macromolecular Architectures Based on Successive Ullmann Couplings

**Figure 5:**
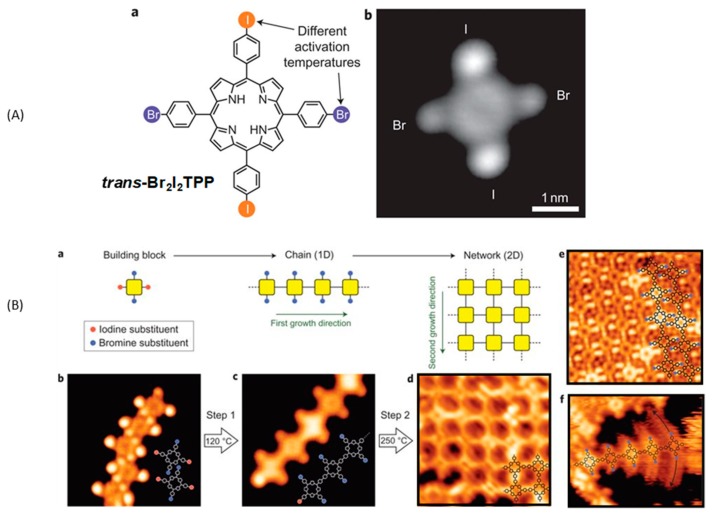
Sequential growth of a 2D architecture with 5,15-*bis*(4′-bromophenyl)-10,20-*bis*(4′-iodophenyl)porphyrin (*trans*-Br_2_I_2_TPP). (**A**) Chemical structure of the precursor. (**B**) Two-step polymer growth. Reprinted by permission from Macmillan Publishers Ltd: Nature Chemistry from [83], copyright 2012.

On-surface chemistry is an emerging field of research and optimization of the reaction conditions to elaborate well-defined nanostructures constitutes the major focus of researchers. Since the first reports mentioning the elaboration of sCOFs, Ullmann coupling of halogenated aromatic rings [31,82] have undoubtedly been one of the most commonly used coupling mode. As mentioned previously, the sequential growth of covalent networks is mainly based on the attachment of two different types of functional groups onto the same monomer. Deviating from this situation, the reactivity of halogens is well known to differ when two different types of halogens are attached to the same molecules. Numerous reactions in solution phase chemistry are based on these differences of reactivity between halogens and this specificity was transposed to on-surface synthesis. In this case, Ullmann coupling can be selectively activated by a careful control of the reaction temperature and fully conjugated 2D polymers can be prepared. This situation typically constitutes a hierarchical growth of 2D structures. In this field, the first report mentioning a hierarchical approach was published in 2012 by Grill et al. [83]. To achieve a complete control during the hierarchical growth, iodine and bromine atoms were introduced onto tetraphenyl porphyrin to give 5,15-*bis*(4′-bromophenyl)-10,20-*bis*(4′-iodophenyl)porphyrin (*trans*-Br_2_I_2_TPP) (see Figure 5A). 

The bond dissociation energy (BDE) of these carbon-halogen bonds are different and determined in the gas phase as being 336 and 272 kJ/mol for the C–Br and C–I bonds, respectively. To anticipate the reactivity of monomers on surface, determination of BDEs in the gas phase are not sufficient. Indeed, the role of the metal surface is crucial (metal choice, crystallographic plane, etc.) and the possibility for the monomers to interact with the substrates are not taken into account in the theoretical calculations. Based on previous works devoted to the Ullmann coupling of halogenated precursors on surface, a scale of reactivity of the carbon–halogen bond could be established, taking in consideration these different parameters. Thus, the C–I bond is well-known to spontaneously cleave on copper, silver, and gold [28,84]. Conversely, the C–Br bond can fully cleave on copper [85,86], partially on silver [87,88], and remains intact on gold [20] in the absence of thermal activation. In this context, the combination of iodine and bromine halogens on *trans*-Br_2_I_2_TPP was appropriate for works carried out on gold. Precisely, the deposition of *trans*-Br_2_I_2_TPP was examined on Au(111) substrates. After deposition of *trans*-Br_2_I_2_TPP at −93 °C, which let the monomer intact, the sequential growth could be activated by heating first the surface at 120 °C and then 250 °C. Here again, the well-separated temperature windows enabled to perfectly control the two-step process and to avoid the possibility of competing Ullmann couplings. As shown in the Figure 5B, the first step produced linear chains of porphyrins by activation of the iodine sites, without forming lateral connection between the different chains. By activating the second growth direction at a higher temperature, a 2D architecture was obtained, resulting from the crosslinking of the different chains. Regular structures extending on surfaces of area bigger than 10 × 10 nm^2^ could be imaged. Catalytic activity of the gold surface in this second step was clearly evidenced as the C–Br bond dissociation could be obtained at 200 °C on the gold surface, below the temperature required to get a bromine dissociation in the evaporator (300 °C) [83]. 

**Figure 6:**
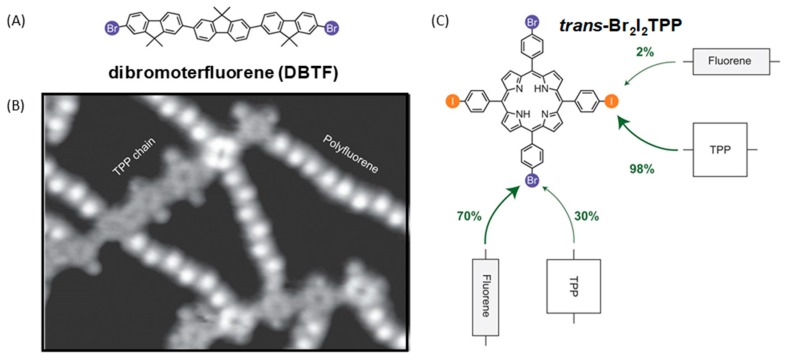
(**A**) Chemical structure of dibromoterfluorene (DBTF). (**B**) STM topograph of the copolymerization of 5,15-*bis*(4′-bromophenyl)-10,20-*bis*(4′-iodophenyl)porphyrin (*trans*-Br_2_I_2_TPP) with DBTF (13 × 18 nm^2^). (**C**) Statistical analyses of the porphyrin/TBTF connection. Reprinted by permission from Macmillan Publishers Ltd: Nature Chemistry from [83], copyright 2012.

**Figure 7:**
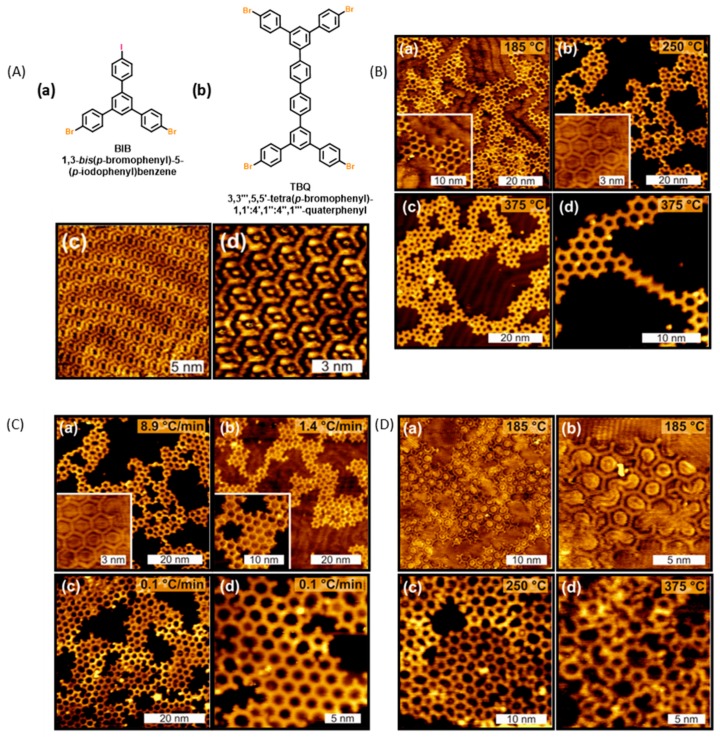
(**Aa**,**b**) Chemical structures of 1,3-*bis*(*p*-bromophenyl)-5-(*p*-iodophenyl)benzene (BIB) and TBQ. (**Ac**,**d**) STM images of supramolecular self-assembly of 3,3‴,5,5′-tetra(*p*-bromophenyl)-1,1′:4′,1″:4″,1‴-quaterphenyl (TBQ) on Au(111) surface. (**B**) STM images of the 2D polymers obtained by annealing the surface at 185, 250, and 375 °C. (**C**) STM images of the 2D polymers obtained by annealing the surface at 250 °C at different heating rates. (**D**) STM images of the 2D polymers obtained by direct polymerization at different temperatures. Reprinted with permission from [89]. Copyright 2014 American Chemical Society.

The possibility to design more complex structures was examined by copolymerizing *trans*-Br_2_I_2_TPP with dibromoterfluorene (DBTF) on Au(111) surfaces (see Figure 6A). Thanks to the sequential procedure, even if both types of molecules are deposited onto the metal surface, heating of the surface at 250 °C first induced the formation of porphyrin chains by activation of the iodine sites (only 2% of undesired reaction of DBTF with *trans*-Br_2_I_2_TPP was detected) followed in second step by the carbon–bromine dissociation resulting in the covalent coupling of the porphyrin chains with DBTF. Chains with length ranging between 15 and 20 nm could be imaged. Even if the lateral connection of adjacent porphyrins was detected on the STM images (see Figure 6B,C), 70% of the carbon–carbon bonds resulted from the reaction between the bromine sites of porphyrin and DBTF, opening the way towards the design of complex architectures. Following this work, the design of 2D polymers with 1,3-*bis*(*p*-bromophenyl)-5-(*p*-iodophenyl)benzene (BIB) was examined (see Figure 7A) [89]. Here again, the two steps are characterized by a significant difference of their activation temperatures since the first step (activation of the iodine sites of BIB) could be realized at room temperature whereas an annealing at 185 °C was required to initiate the second step (activation of the bromine sites of BIB). As anticipated, deposition of BIB on Au(111) surfaces at room temperature resulted in the dimerization of BIB and the formation of 3,3‴,5,5′-tetra(*p*-bromophenyl)-1,1″,1″:4″,1‴-quaterphenyl (TBQ). STM images of the supramolecular phase revealed the TBQ units to be linearly aligned in parallel rows, with iodine atoms standing between the TBQ motifs.

The influence of the reaction temperatures (185, 250 and 375 °C) and the heating rates (between 6.3 and 8.9 °C/min) on the second step were examined. Irrespective of these two parameters, a similar disorder accompanied with an incomplete coverage of the surface and the formation of pores could be found on all STM images, of scanning size 60 × 60 nm^2^ for the bigger STM images (see Figure 7B). Complex networks based on highly branched structures could be imaged. As interesting findings, hexagons and opened pores were mainly observed upon annealing the surface at 185 °C whereas squares and octagons were more frequently observed at 250 and 375 °C (see Figure 7C,D). 

**Figure 8:**
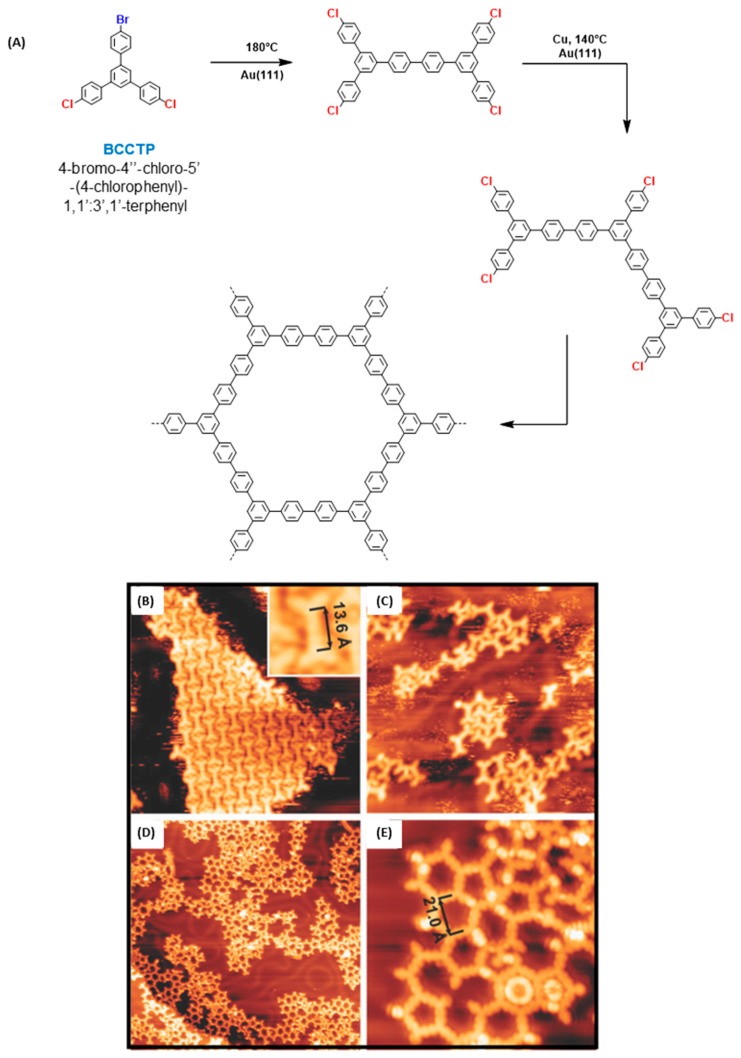
(**A**) Reaction pathway involved in the formation of hexagonal pores with BCCTP. (**B**) Evaporation of BCCTP on Au(111) substrates (26 nm × 26 nm with inset 3 nm × 3 nm). (**C**) Evaporation of the copper catalyst on a Au surface heated at 140 °C (26 nm × 26 nm). (**D**,**E**) STM images of the Au(111) substrate after annealing the surface at 280 °C at different magnifications (65 nm × 65 nm for (**D**) and 14 nm × 14 nm for (**E**)). Adapted from [90] with permission from The Royal Society of Chemistry.

Parallel to the hierarchical polymerization, the possibility of creating regular 2D polymers in one step, by direct polymerization, was examined. In this second approach, temperature of the substrates is determinant since 2D polymers of low quality were obtained for surfaces held at 185 and 375 °C whereas regular hexagonal pores were obtained at 250 °C (see Figure 7D). Almost defect-free polymers extending over surfaces of area bigger than 30 × 30 nm^2^ were detected. At such high temperatures, the deposition rate is another parameter affecting the network quality and reduction of the rate resulted in a broader distribution of polygons going from squares to octagons. From these different experiments, it could be concluded that at low temperature, the mobility of molecules was insufficient and the cleavage of C–Br bonds incomplete so that it could account for the large amount of opened pores. Conversely, at high temperature, the probability to generate pores deviating from the ideal hexagons was greatly enhanced. Finally, comparison of the direct and the hierarchical polymerization revealed the first approach to produce a denser polymeric network even if the density of defects was similar in the two cases. Two years later, other authors examined the covalent coupling of 4-bromo-4″-chloro-5′-(4-chlorophenyl)-1,1′:3′,1′-terphenyl (BCCTP) which differs from BIB by the choice of the halogens (see Figure 8A) [90]. 

In this last case and due to the low reactivity of the chlorine atoms, presence of Cu catalyst was required to induce a surface-assisted Ullmann coupling. This is among the first example of Ullmann coupling where a catalyst was introduced to enforce the reaction to occur. As anticipated, and due to the inexistent reactivity of the chlorine atoms, the first step furnished on Au(111) substrates a supramolecular arrangement comparable to that obtained with BIB, with the formation of BCCTP dimers linearly aligned in parallel rows, with bromine atom standing between dimers (see Figure 8B). By evaporating the Cu catalyst on the Au(111) surface kept at 140 °C, the second coupling mode could be activated, enabling the formation of oligomers (see Figure 8C). However, if the polymerization of BCCTP could starts at 140 °C, this latter could only be ended by annealing the surface at 280 °C for 30 minutes (see Figure 8D,E). Once again, a large diversity of polygons was detected on the surface, going from pentagons to heptagons, demonstrating the difficulty to elaborate regular pores. For comparison, a worse result was obtained while depositing BCCTP on Cu(111). Indeed, no control of the polymerization process was possible on this metal surface. Considering that the sequential polymerization of BIB and BCCTP should produce exactly the same final polymers, that the crystallographic plane and the metal chosen for the two studies are the same, and with regards to the size of the STM images (35 × 35 nm^2^ for the polymerization of BIB (see Figure 7(Dc)), 65 × 35 nm^2^ for the polymerization of BCCTP (see Figure 8D), the most regular structures are clearly obtained with BIB which is also the monomer exhibiting the best design to get an advanced polymerization process. In 2017, an interesting study demonstrated the possibility to elaborate a hierarchical growth of 2D polymers with a precursor only bearing one type of halogens [91]. This strategy is quite unexpected considering that the six bromine atoms of 1,3,5-*tris*(3,5-dibromophenyl)benzene (TDBB) could theoretically cleave simultaneously upon thermal activation.

**Figure 9:**
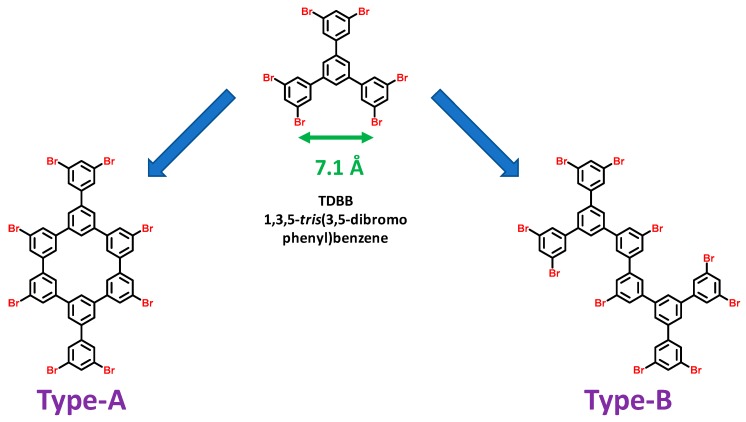
Scheme of the 1,3,5-*tris*(3,5-dibromophenyl)benzene (TDBB) dimer building block with the two-covalent-bond dimer (type-A) and the one-covalent-bond dimer (type-B).

In fact, authors demonstrated this precursor to possibly give rise to two different types of dimers, depending of the coupling modes (type-A and type-B, see Figure 9A–C). 

**Figure 10:**
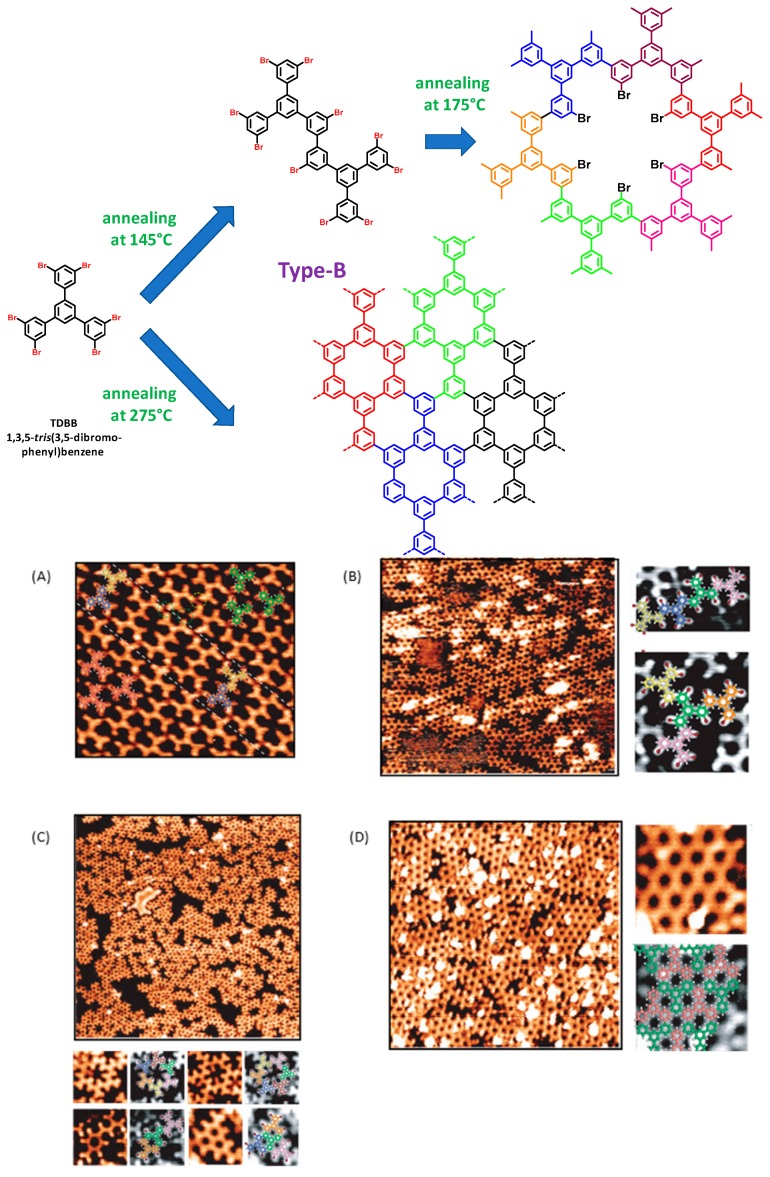
Sequential growth of 2D polymers based on 1,3,5-*tris*(3,5-dibromophenyl)benzene (TDBB). (**A**) STM images of the Au(111) surface after annealing at 145 °C (9 nm × 9 nm). (**B**) STM images of the Au(111) surface after annealing at 170 °C (25 nm × 25 nm). (**C**) STM images of the Au(111) surface after annealing at 175 °C (36 nm × 36 nm and 5 nm × 5 nm (bottom left), 3 nm × 3 nm (bottom right)). (**D**) STM images of the Au(111) surface after annealing at 275 °C (left: 20 nm × 20 nm, right: 3 nm × 3 nm). Adapted with permission from Peyrot at al. [91]. Copyright 2017 American Chemical Society.

In this work, we succeeded in developing temperature-dependent engineering of 2D polymers. Notably, by annealing at 145 °C the supramolecular phase obtained after evaporation of TDBB on Au(111) surfaces—only type-B dimers could be found on the surface—resulting from the formation of a unique type of C–C bond between TDBB (see Figure 10A). Interestingly, rows of type-B dimers were found to be separated from each other by nanodomains only composed of unreacted TDBB. On the opposite, by annealing the supramolecular phase at 170 °C, oligomers of TDBB formed, resulting from multiple type-B couplings (see Figure 10B). 

Locally, star-shaped tetramers could even be found, still resulting from type-B couplings. Regarding their specificity, these tetramers are issued from the covalent linkage of a central TDBB unit surrounded by three peripheral TDBB. By annealing the supramolecular phase at 175 °C (i.e., at a temperature only higher of 5 °C compared to the previous experiments), the coexistence of different structures could be found, as exemplified in the Figure 10C. Notably, cyclic structures composed of six molecules connected by type-B linkages could be found all over the surface. More scarcely, structures based on type-A couplings could also be found. However, irrespective of the type of linkage, the formation of a dense polymeric structure with dimensions bigger than 40 × 40 nm^2^ (i.e., the size of the STM images) can be detected on Figure 10D, demonstrating the efficient of the Ullmann coupling on Au(111) surfaces. Finally, annealing of the supramolecular phase at 275 °C enabled to form a densely packed 2D networks with hexagonal pores exclusively based on type-A linkages (see Figure 10D). Regularity of the final polymers could be evidenced with a scan area size larger than 20 × 20 nm^2^. 

From these different experiments, the sequential growth of the 2D polymers could be deduced. Thus, annealing of the supramolecular phase resulted in first step in the dimerization of TDBB via a single type-B connection. Starting at 170–175 °C, the covalent linkage of three (or more) TDBB units by mean of two type-B connections occurs, giving rise to the formation of linear chains. By increasing the temperature until 275 °C, connections between chains can be initiated, inducing the formation of type-A linkages between molecular tectons. In turn, due to its stepwise growth, a porous network exhibiting a low defect density can be obtained.

### 2.2. Sequential Growth Based on the Boroxine Formation/Ullmann Coupling Combination

**Figure 11:**
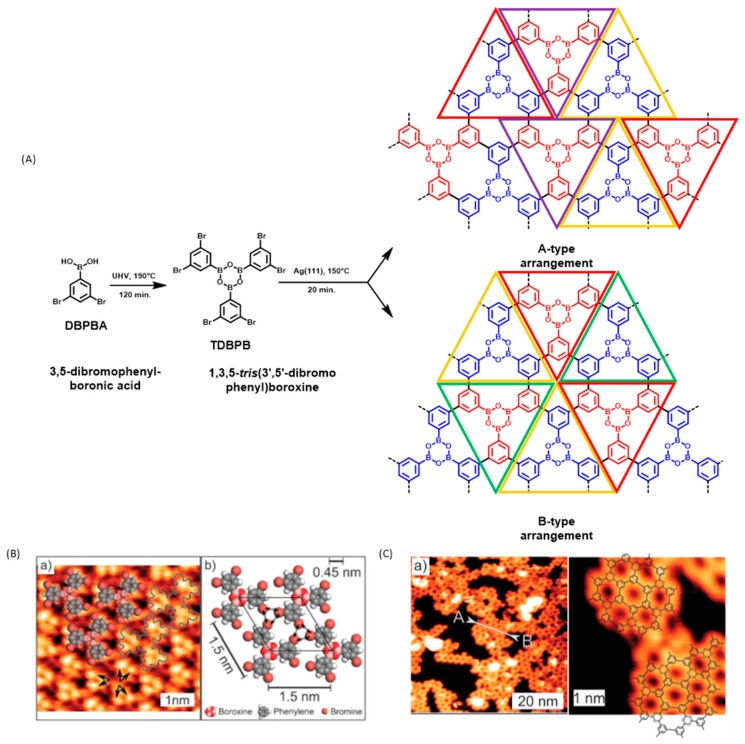
Reactions involved in the polymerization process of 3,5-dibromophenylboronic acid (DBPBA). (**A**) The two potential A- and B-type arrangements. (**B**) STM topograph of the supramolecular phase on Ag(111) obtained with 1,3,5-*tris*(3′,5′-dibromophenyl)boroxine (TDBPB) and the tentative model of the monolayer. (**C**) STM topographs of the polymer phase at different magnifications. Reproduced from [94] with permission from The Royal Society of Chemistry.

Since the first reports mentioning the elaboration of sCOFs, condensation of boronic acids to form boroxines [92,93] and the Ullmann coupling of halogenated aromatic rings [31,82] have been widely used as coupling modes. Considering that these two on-surface reactions are now a mature field of research with regards to the number of publications, a combination of these two reactions was logical to create 2D covalent networks. The first report mentioning such a combination was published in 2011 [94]. First of all, orthogonality of the two reactions is obvious and no side-reactions are expected to occur at each step. 3,5-Dibromophenylboronic acid (DBPBA) was selected as the molecular tecton and choice of the boronic functional group for DBPBA was dictated by the fact that the boroxine cycle that results from the trimerization process of boronic acids exhibits the same symmetry and almost the same size than a phenyl ring, while sharing its planarity. Under UHV and upon deposition onto Ag(111) substrates, dehydration of DBPBA could be initiated at low temperature produced the unreactive 1,3,5-*tris*(3′,5′-dibromophenyl)boroxine (TDBPB) (see Figure 11). It has to be noticed that the dehydration reaction of boronic acids possesses its own specificity as this reaction does not require the presence of a catalyst and directly occurs during heating in the evaporator. Indeed, upon heating, condensation of boronic acids is preferred over sublimation. In situ formation of TDBPB was confirmed by Raman spectroscopy and by NMR analyses. An additional proof of the formation of trimers was obtained by sublimation of DBPBA onto graphite(001) substrates; only the presence of TDBPB was detected on the STM topographs. No trace of DBPBA was observed on the surface, demonstrating that the cyclodehydration of boronic acids was quantitative in the evaporator. Stability of the supramolecular phase is ensured by electrostatic interactions between halogens, assembled into trigonal cyclic arrangements (see Figure 11A). Interestingly, annealing of the graphite(001) substrates at 200 °C only resulted in the desorption of the molecules whereas annealing of the Ag(111) substrates at lower temperature (130 °C) furnished the targeted polymer by a surface-catalyzed reaction. The higher ability of silver to interact with organic molecules than graphite is a general trend reported in numerous works concerning the on-surface synthesis and the better adsorption of TDBPB on Ag(111) substrates over graphite substrates confirms this trend. Due to the specific substitution of DBPBA (and therefore of TDBPB), the coexistence of two hexagonal arrangements (A-type and B-type) could be potentially observed on the surface (see Figure 11A,B). However, the periodicity of the final network, the distance between cycles, and the symmetry of the overall system revealed the exclusive formation of A-type arrangements. As a drawback, the final polymer lacks long-range order and only small nanodomains were detected on the surface (see Figure 11C). Indeed, nanodomains rarely bigger than 20 × 20 nm^2^ could be found on the surface. The low polymerization yield can be confidently assigned to the molecularity of the reaction in the second step, six functional groups being simultaneously involved in the Ullmann coupling of TDBPB. This issue was addressed in the next study where the number of halogens were reduced by a factor 2 compared to this study [95]. Similarly to DBPBA, the formation of hexagonal pores resulted from the combination of two consecutive steps, the building block being this time *p*-bromobenzeneboronic acid (BBBA). Due to its reduced molecular weight, the sublimation of BBBA onto Au(111) substrates could be realized at lower temperature than that required for the trimer of DBPBA (100 °C for BBBA vs. 190 °C for the trimer of DBPBA). 

Compared to DBPBA, the advantages offered by BBBA are manifold: (1) The low molecular weight of BBBA drastically limits the degradation and the probability of polymerization during sublimation. In fact, investigations revealed BBBA to be deposited intact on the Ag(111) substrates and to polymerize subsequent to its deposition on the Au(111) surface. The competition exiting between sublimation and polymerization for boronic acids is a well-known drawback of this family of compounds since only a minor fraction of boronic acids can be sublimed onto the surface, the rest polymerizing in the evaporator. (2) Limitation of the molecularity of the reaction at each step optimizes the probability of reaction and thus improves the reaction yields. Possibilities to create defects are considerably reduced. (3) Creation of hexagonal pores of large size becomes possible, the two-step growth enabling to avoid the evaporation of large precursors with important molecular weight. However, the detrimental role of the intermediate supramolecular phase was clearly evidenced. 

**Figure 12:**
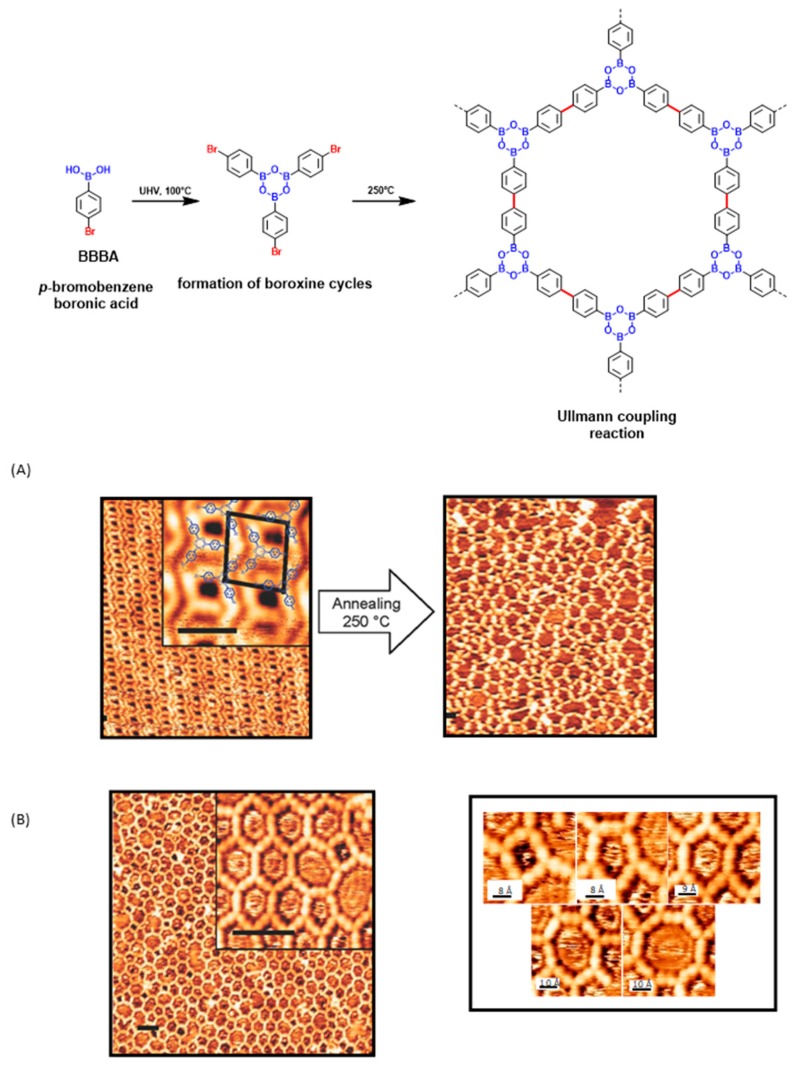
Polymerization reaction of *p*-bromobenzeneboronic acid (BBBA) on Au(111) substrates. (**A**) STM topographs of the supramolecular phase further annealed at 250 °C (10 nm × 14 nm, inset: scale bar = 2 nm). (**B**) STM topograph of the polymerization of BBBA in one step. Polygons of different shapes found on the Au(111) surface (left: scale bar = 4 nm). Adapted with permission from [95]. Copyright 2012 American Chemical Society.

Due to the formation of dense nanodomains of BBBA dimers stabilized by hydrogen bonds, the distance between trimers was not sufficient for the molecules to rearrange and diffuse onto the surface during annealing at 250 °C. If the formation of the polymer network was clearly evidenced, the reaction yield was limited to 88%, insufficient for the ring-closure of hexagons. Only 40% of pores covering the surface were detected (see Figure 12A).

In fact, investigations revealed the density of BBBA dimers in the supramolecular phase to be 1.5 BBBA/nm^2^, higher than the density of BBBA in an ideal honeycomb-like structure (1.2 BBBA/nm^2^). The material being denser in the supramolecular phase than in the polymer network, it clearly supports the formation of numerous defects during the conversion of the supramolecular assembly to the polymer. As a consequence of the high density of molecules on the surface and the insufficient distance between molecules to rearrange, polygons of different shapes could be detected, going from squares to octagons. Defects could not be repaired, even by a post-thermal treatment of the surface at 400 °C. The drawback issued from the close packing of the molecules in the supramolecular phase is an issue that has already been reported in the literature [29,96]. To address the density issue, BBBA was directly deposited on Au(111) substrates maintained at 250 °C. In these conditions, the two steps could be realized at low molecular density, favoring the diffusion and the rotation of the molecules on the surface and enabling in turn the molecules to be properly positioned for the formation of the boroxine cycles and the Ullmann couplings (see Figure 12B). As evidenced in the Figure 12B, an STM image with a scan area size larger than 55 × 55 nm^2^ revealed the surface to be totally covered by the polymer, demonstrating that the polymerization can be carried out over a large area. A polymerization yield increasing up to 95% accompanied with the formation of 60% of pores were determined. Compared to the postannealing approach (first attempt), deposition of BBBA at high temperature favored the polymerization process. The total area covered by the polymer was doubled compared to the first attempts, even if a similar distribution of pore shapes was found in the two cases. Disorder in the final polymer was assigned to the size of the BBBA trimer, favoring the flexibility of the structure and the deviation from the regular hexagons. These conclusions are consistent with previous results reported in the literature for monomers of equivalent size [20,86]. Finally, examination of metal surfaces other than Au(111) revealed the choice of the surface to be essential to get well-separated temperature windows for the two steps. Thus, experiences carried out on Ag(111) revealed the two reactions to occur simultaneously, resulting in the formation of disordered structures on the surface. 

### 2.3. Sequential Growth Based on the Boroxine Formation/Imine Combination

**Figure 13:**
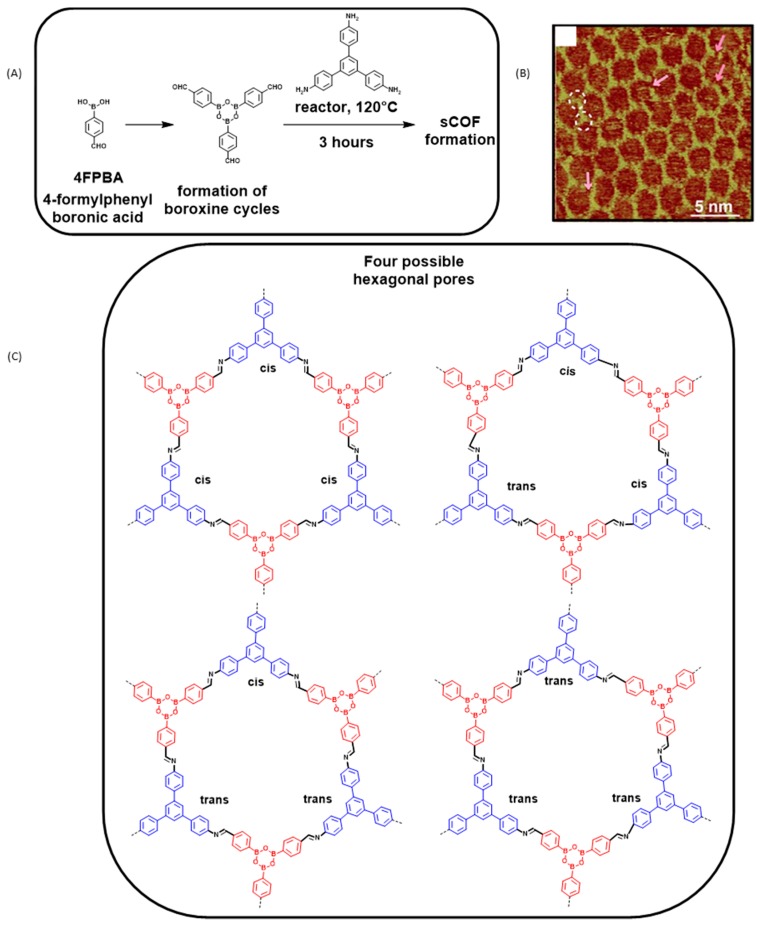
Polymerization reaction of 4-formylphenylboronic acid (4FPBA) with 1,3,5-*tris*(4-aminophenyl)benzene (TAPB). (**A**) The two reaction steps. (**B**) STM topograph of the polymer network. (**C**) the different possible conformations of the imine groups and the resulting hexagons. Reproduced from [97] with permission from The Royal Society of Chemistry.

Capitalizing on the findings of the former studies, the possibility to combine two orthogonal reactions, namely, the formation of the boroxine cycles and imines in a single step was examined in 2016 on highly oriented pyrolytic graphite (HOPG) (see Figure 13) [97]. As the main difference from the previous works, the formation of sCOFs was examined in a multicomponent reaction with the amine and the aldehyde functional group attached to different precursors. Influence of the substitution pattern was also studied by differing the position of the aldehyde functional group. 

As main advantages of these selected reactions, even if the two reaction pathways are activated simultaneously, the boroxine and imine formations are reversible reactions based on the release of water molecules which is the same side-product for the two reactions. In this context, self-healing opportunities during the polymerization process are greatly improved by the presence of numerous water molecules. With reference to “chemically released diffusion”, self-healing typically refers to this concept, the possibility to repair defects resulting from mass transfer between polymer chains under growth. Considering that water molecules are exchanged between macromolecules under growth enabling to cleave and reform specific bonds, the term “chemically-assisted healing” would be more accurate in the present case. 

To form the 2D structures, two bifunctional precursors were examined, namely 4-formylphenylboronic acid (4FPBA) and 3-formylphenylboronic acid (3FPBA) that were both opposed to 1,3,5-*tris*(4-aminophenyl)benzene (TAPB). Interestingly, two different chiral phases were obtained upon reaction of 3FPBA with TAPB and these results are among the first examples of chiral sCOF reported in the literature (see Figure 13). To perform these different two-component reactions, TAPB was first drop-casted onto the HOPG substrate whereas the second partner (4FPBA or 3FPBA) was placed in a reactor as powder in the presence of copper sulfate pentahydrate as the equilibrium control agent [92].

When 4FPBA was used as the precursor, three hours at 120 °C in a sealed reactor were required to obtain a polymerization. Typically, nanodomains of 80 × 80 nm^2^ were detected, the growth of these domains being orientated along the lattice of the HOPG surface (see Figure 13B). Due to the possible cis/trans isomerization of the imine functions, an inherent disorder exists and the presence of distorted hexagons on the surface is clearly detected (see Figure 13C). As the main drawback of employing functional groups with a nonpermanent orientation, presence of unclosed hexagons was observed on the HOPG substrate due to the inappropriate orientation of numerous imine functional groups.

**Figure 14:**
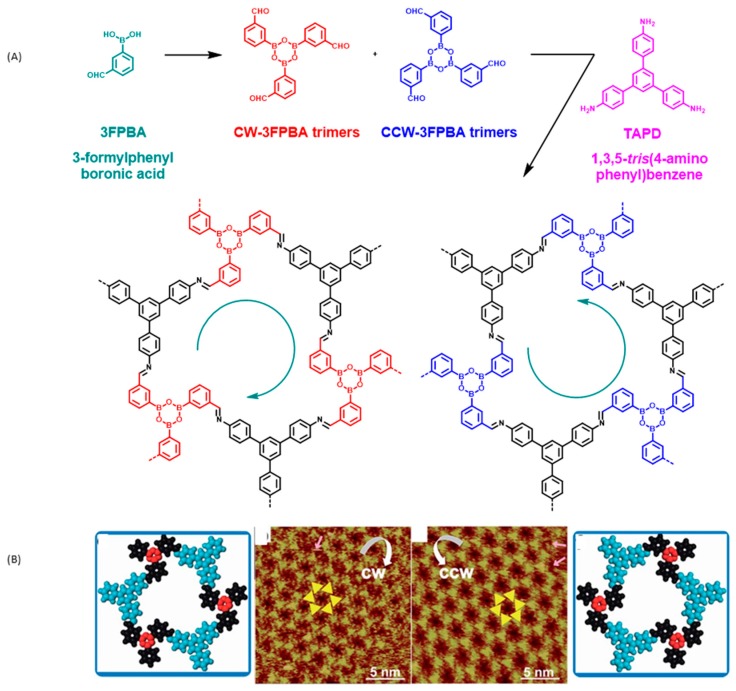
(**A**) Reaction involved in the polymerization process. Clockwise (CW) and counter-clockwise (CCW) rotating 3FPA trimers. (**B**) STM topographs of the sCOF obtained with 3-formylphenylboronic acid (3FPBA) and 1,3,5-*tris*(4-aminophenyl)benzene (TAPB), and the demonstration of the presence of two chiral phases. Reproduced from [97] with permission from The Royal Society of Chemistry.

A more complicated behavior was evidenced for 3FPBA. Indeed, due to the asymmetrical substitution of 3FPBA, a chirality can appear due to the formation of two types of 3FPBA trimers that can rotate clockwise (CW-3FPBA) or counterclockwise (CCW-3FPBA) (see Figure 14A). By polymerization with TAPB, the coexistence of two chiral nanodomains with a long-range order was found on the surface) (see Figure 14B). As specificity, CW-3FPBA furnished a CCW-sCOF, whereas the opposite situation was found for CCW-3FPBA (CW-sCOF). For the two chiral sCOFs, a growth orientation governed by the interaction of TAPB with the substrate was found, imposing the epitaxial conditions of growth. 

**Figure 15:**
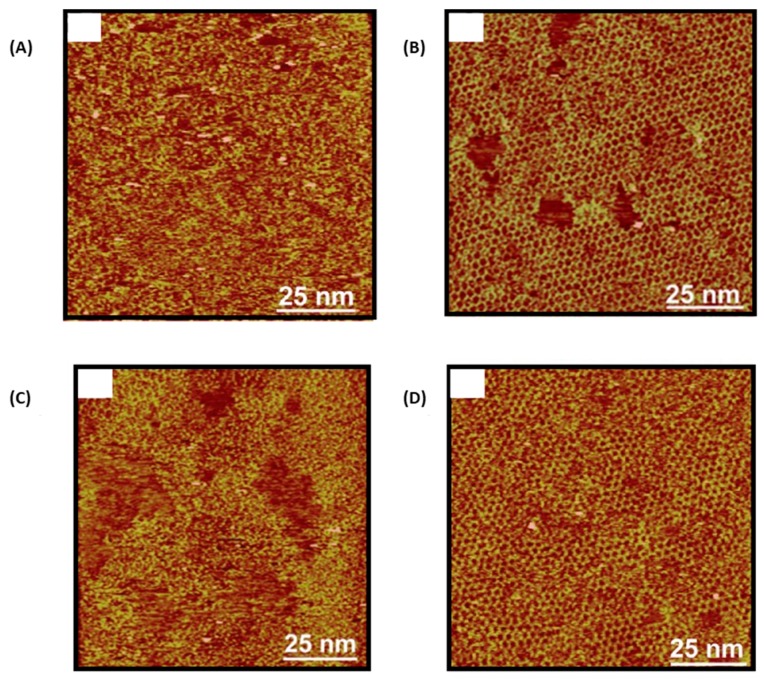
(**A**) STM topograph of the polymerization of 4-formylphenylboronic acid (4FPBA) with 1,3,5-*tris*(4-aminophenyl)benzene (TAPB) without copper sulfate pentahydrate. (**B**) STM topograph of the polymerization of 4FPBA with TAPB for a reaction carried out at 120 °C for 3 h. (**C**) Stability of the 2D polymer formed with 4FPBA and TAPB after heating at 180 °C for 1 h. (**D**) Stability of the polymer formed from 4FPBA and TAPB after 20 days of storage. Reproduced from [97] with permission from The Royal Society of Chemistry.

Evidence of imines formation was furnished by X-ray photoelectron spectroscopy (XPS) analyses. A peak at 398.5 eV characteristic for the nitrogen of an imine group was detected in the XPS spectrum. Conversely, a peak at 399.8 eV was found for the precursor TAPB, demonstrating the chemical modification of the NH_2_ group. The crucial role of copper sulfate pentahydrate was clearly demonstrated in control experiments. Notably, a higher structural disorder accompanied by incomplete reactions was found for the polymerization of 4FPBA with TAPB in the absence of this salt (see Figure 15A). 

In fact, this hydrated salt is capable in a sealed reactor to release water and favor the reversibility of the imine and boroxine formation. Examination of the kinetic of polymerization of 4FPBA with TAPB also revealed the boroxine cycle to be formed at the same timescale than the imine functional groups, and that a high coverage of the HOPG surface can only be obtained after four hours of reaction (see Figure 15B). Finally, stability of the sCOFs were examined and several conclusions could be determined. Thus, sCOFs formed with 4FPBA were stable after annealing of the surface for one hour at 180 °C, demonstrating the robustness of the structure (see Figure 15C). Second, after 20 days of storage under ambient conditions, sCOFs formed with 4FPBA could be still observed on the substrates what is remarkable considering that the boroxine cycle is easy to hydrolyze (see Figure 15D). This stability is higher than that previously reported for sCOF only formed of boroxine cycles [92]. However, examination of the chemical stability of these structures revealed that exposition of sCOF to acidic (pH < 3) or basic (pH > 11) conditions rapidly furnished chaotic structures.

### 2.4. Hierarchical Growth Based on the Coordination Polymer/Phthalocyanine Formation

**Figure 16:**
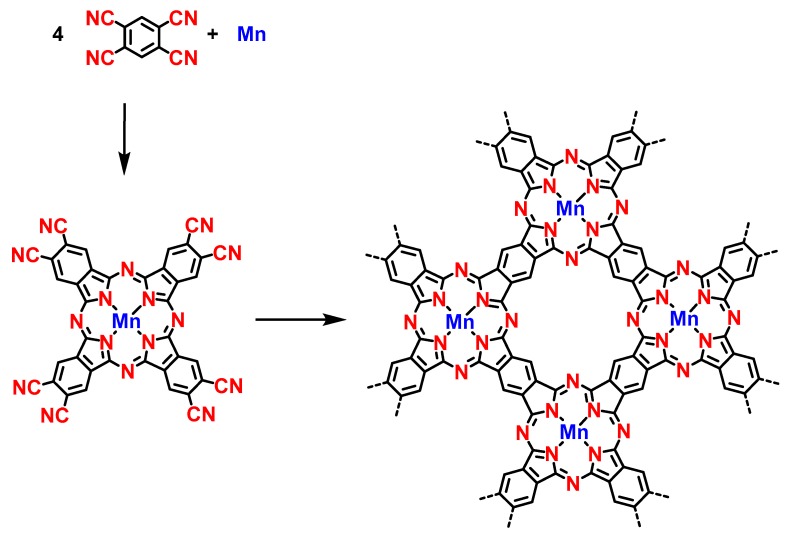
Sequential growth of the phthalocyanine-based polymer.

**Figure 17:**
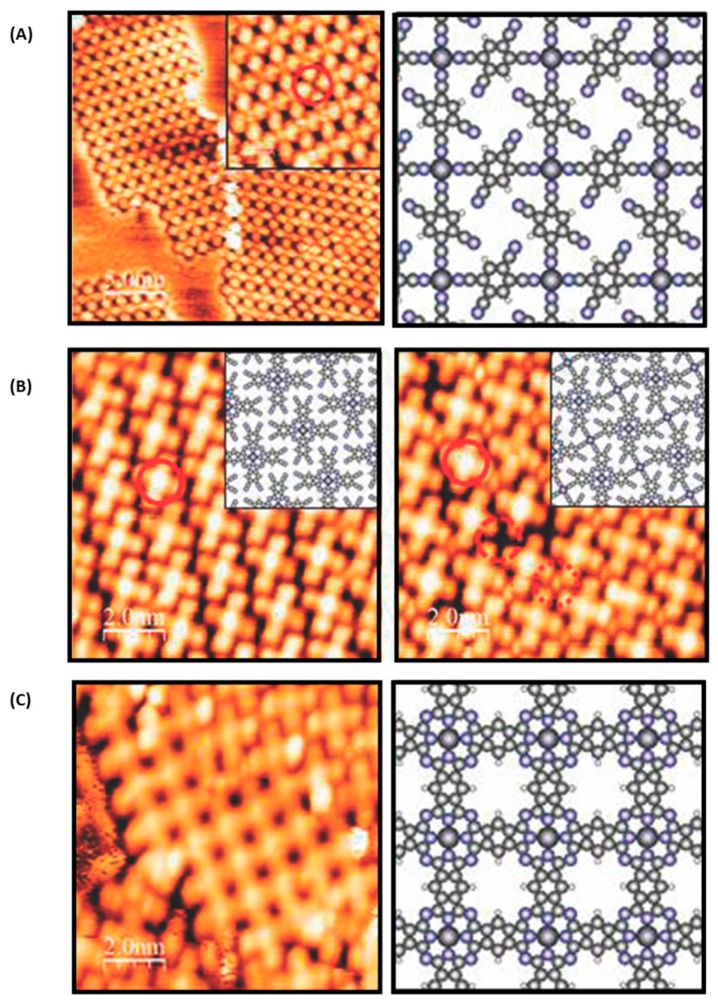
The different steps involved in the formation of phthalocyanines-based 2D π-conjugated polymers. (**A**) STM images of the supramolecular phase resulting from the deposition of 1,2,4,5-tetracyanobenzene (TCNB) and Mn atoms on a Ag(111) surface at room temperature with a DFT model. (**B**) STM images of the two phases existing after annealing at 277 °C. Left: the hydrogen-bonded supramolecular phase. Right: the metal–organic coordination 2D polymers. (**C**) Polymeric phase obtained in the final step upon annealing at 342 °C. Reproduced from [98] with permission from The Royal Society of Chemistry.

A longstanding challenge toward the development of advanced functional materials relies in the possibility to create fully conjugated covalent networks. An interesting approach consisted of creating in situ the phthalocyanine macrocycles in successive steps involving first the formation of a supramolecular phase further converted to a covalent polymer by thermal annealing [98]. Phthalocyanines are a family of compounds extensively used in organic electronics by the possibility to finely tune their electronic properties by a careful selection of the metal center. These metal complexes are also characterized by a remarkable thermal and chemical stability, making these structures appealing candidates for numerous applications. Parallel to this, numerous examples of on-surface chemistry based on the phthalocyanine motif have been reported in the literature [99]. Here, and contrarily to the former study reported in 2011 where a polymer of Fe-phthalocyanine was formed in one step on Au(111) [100], in this subsequent study carried out by the same authors, the different intermediate steps could be isolated and characterized (see Figure 16). As the main difference, Mn instead of Fe was used as the metal, Ag(111) substrate was used instead of Au(111), while 1,2,4,5-tetracyanobenzene (TCNB) was used as the same elemental building block. In this work, formation of the phthalocyanine polymer could be decomposed in three steps, the first one consisting in the sublimation of both the metal and the precursor on Ag(111) held at room temperature, generating a two-dimensional coordination polymer with a square symmetry (see Figure 17A). In this first 2D network, Mn and TCNB could be found in a 1:1 ratio. Upon annealing at ca 377 °C, Mn-based octacyanophthalocyanines arranged in a closely packed supramolecular structure self-assembled by hydrogen bonds and aligned along the dense directions of the substrate were found (see Figure 17B).

Coexisting with this first phase, a second phase, where Mn-based octoacyanophthalocyanines are linked by mean of Mn atoms, could also be found. Considering that Mn-based octoacyano-phthalocyanines can be formed in a 4:1 TCNB:Mn ratio, the presence of this second phase resulting from metal–ligand interactions between the electron-donating nitrile groups and the electron-deficient metal atoms could be attended. It also supports the formation in the final stage (annealing at 342 °C) of small 5-nm-large domains composed of polymeric phthalocyanines, where the presence of free Mn atoms is necessary to form the additional phthalocyanines and form the fully conjugated polymer (see Figure 17C). 

In this 2D polymeric phase, magnetic atoms regularly spaced and introduced in a fully π-conjugated system could be obtained. This work opens the way towards the bottom-up elaboration of devices for data storage.

## 3. Coupling Modes Used for the Design of 1D Macromolecular Organic Structures

### 3.1. Sequential Growth Based on the Ullmann Coupling/Aromatization Combination

**Figure 18:**
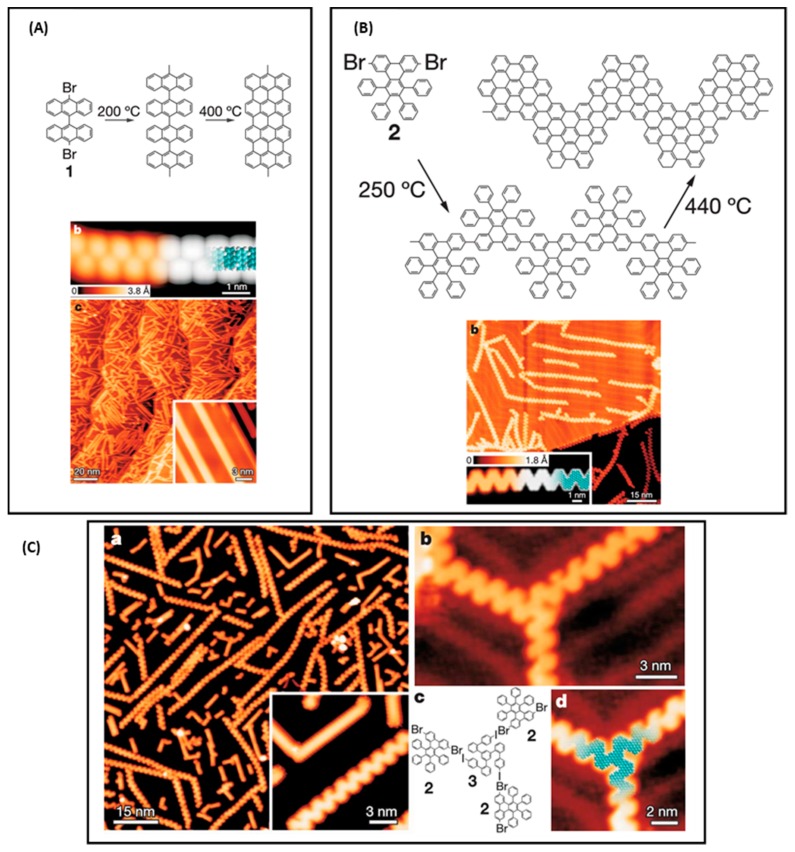
(**A**) Reaction mechanism and STM images of the formation of nanoribbons with 10,10′-dibromo-9,9′-bianthracene. (**B**) Reaction mechanism and STM images of the formation of zigzag nanoribbons with 6,11-dibromo-1,2,3,4-tetraphenyltriphenylene. (**C**) STM images of the copolymerization reaction. All experiments have been carried out on Au(111) surfaces. Reprinted by permission from Macmillan Publishers Ltd: Nature from [101], copyright 2010.

In all the aforementioned reaction combinations, 2D covalent networks have been obtained by sequential or hierarchical growth of nanostructures. However, a series of combinations has also been specifically developed for the design of 1D macromolecular organic structures. At present, it has to be noticed that these combinations have not been used for the design of 2D structures yet, even if from a technical point of view, these combinations could be easily transposed to 2D structures. In this field, graphene, by its unique properties, has driven a great deal of interest and numerous works have been devoted to design graphene-like structures. To produce regular and extended structures, the Ullmann coupling followed by an aromatization reaction is a promising approach (see Figure 18).

**Figure 19:**
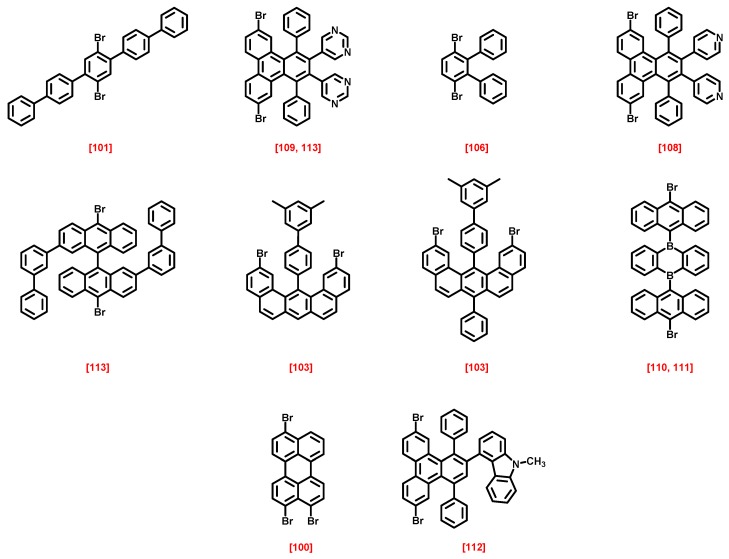
Chemical structures of precursors used to elaborate graphene nanoribbons (GNRs) and the corresponding reference in brackets.

In this field, the first on-surface synthesis was reported in 2010 [101]. Graphene nanoribbons (GNRs) were obtained by first depositing 10,10′-dibromo-9,9′-bianthracene on Au(111) surfaces at 200 °C, enabling to interlink the biradicals resulting from the dehalogenation reaction, the planarization of the structure and the formation of a linear polymer. At 400 °C, a cyclodehydrogenation reaction converted the polymer to a fully conjugated and linear structure (see Figure 18A). Versatility of the approach was demonstrated by the design of several types of nanoribbons, such as chevron-type nanoribbons resulting from the polymerization and aromatization reaction of 6,11-dibromo-1,2,3,4-tetraphenyltriphenylene (see Figure 18B). In this last case, steric hindrance generated by the tetraphenylene groups enforces these bulky substituents to stand on each side of the polymer main axis and a zigzag structure was obtained. The possibility of codepositing two differing precursors (6,11-dibromo-1,2,3,4-tetraphenyltriphenylene and 1,3,5-*tris*(4”-iodo-2′-biphenyl)benzene) was also examined, demonstrating the possibility to design chemically modified graphene-like structures (see Figure 18C). Recently, the different experiments carried out by Cai et al. on 10,10′-dibromo-9,9′-bianthracene and 10,10′-dibromo-9,9′-bianthracene were reproduced using another deposition process for the first step. In this last case, the direct contact transfer (DCT) consisting in using a stamp with the corresponding molecule at its surface was used [102]. Using this strategy, the precursor could be deposited on the surface without taking recourse to sublimation. This finding constitutes a major advancement, considering that the molecular weight often constitutes a drastic limitation. Indeed, increase of the molecular size results in higher sublimation temperatures that can degrade the molecule. Over the years, a wide range of molecular tectons giving access to GNRs have been reported in the literature and these different structures are depicted in the Figure 19 [103,104,105,106,107,108,109,110,111,112,113,114,115,116,117]. 

**Figure 20:**
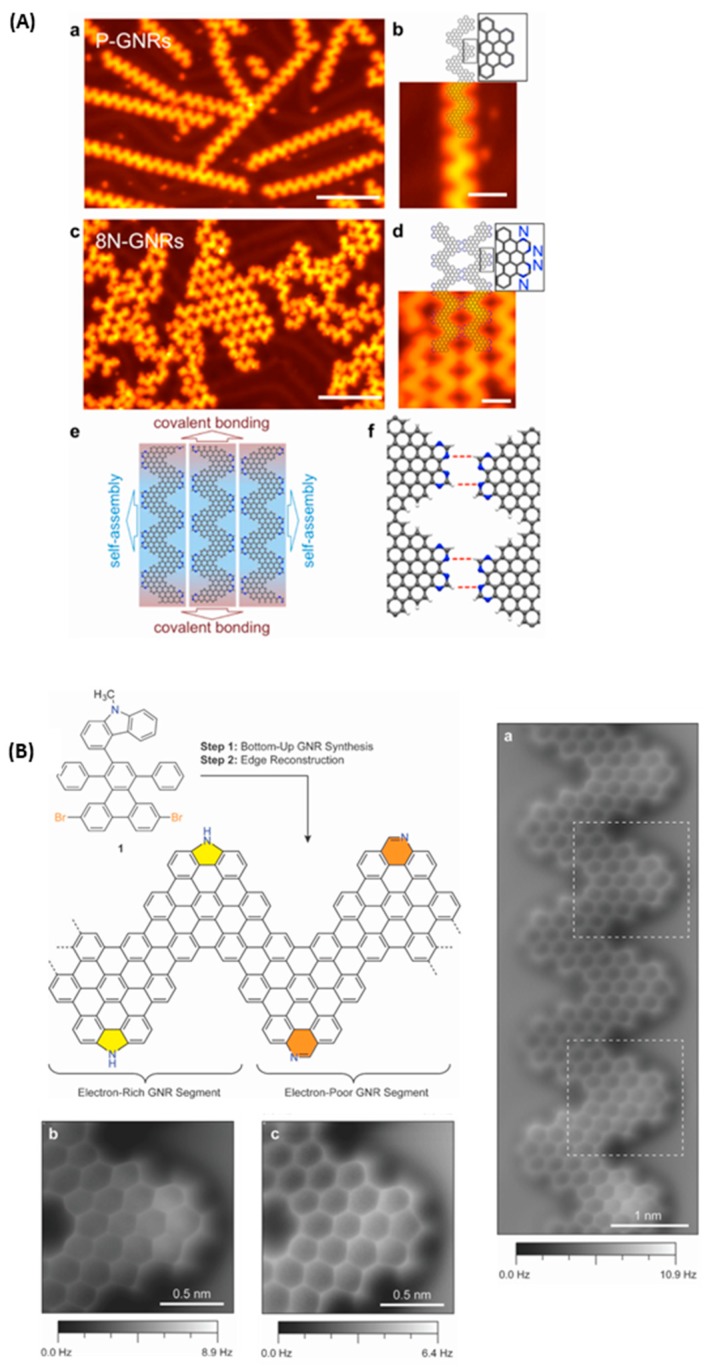
(**Aa**–**f**) Bottom-up synthesis of GNRs containing nitrogen atoms with the possibility to create lateral interactions between GNRs (a: scale bar: 10 nm, b: scale bar: 2 nm, c: scale bar: 10 nm, d: scale bar: 2 nm). Adapted with permission from Vo et al. [112]. Copyright 2015 American Chemical Society. (**B**) Schematic representation of GNRs comprising both carbazole and phenanthridine units. (**Ba**–**c**) High-resolution AFM images of GNRs with the demonstration of the presence of phenanthridine (**Bb**) and carbazole (**Bc**) units in GNRs. Reproduced from [113] with permission from The Royal Society of Chemistry.

The beauty of these approaches relies in the fact that nitrogen-doped GNRs [111,116], ultranarrow GNRs [103,118], chevron-type GNRs [102], or zigzag GNRs [106,117,119] with chemically modified edges could be elaborated with these precursors. Among the most interesting findings and by properly choosing the molecular precursors, GNRs could notably be used as elemental building blocks to trigger the design of more extended structures. This goal was achieved by substituting the precursors with nitrogen atoms [112]. Ordered nanomaterials could be obtained by an atomically precise bottom-up synthesis based on the creation of hydrogen bonds and van der Waals interactions between GNRs (see Figure 20A). Growth of structures in the third dimension could be even obtained by π–π stacking of GNRs sheets. Modification of the electronic structures of GNRs is another long-standing challenge and, in this field, a remarkable example has been reported in 2016 [115]. 

By a thermally activated ring expansion/dehydrogenation reaction, the electron-rich carbazole could be converted into the electron-deficient phenanthridine [120]. In this work, authors succeeded to partially convert the carbazole groups into phenanthridine, opening the way towards the fine tuning of the materials bandgaps by the presence of electron-rich and electron-poor groups onto the same structures (see Figure 20B). 

**Figure 21:**
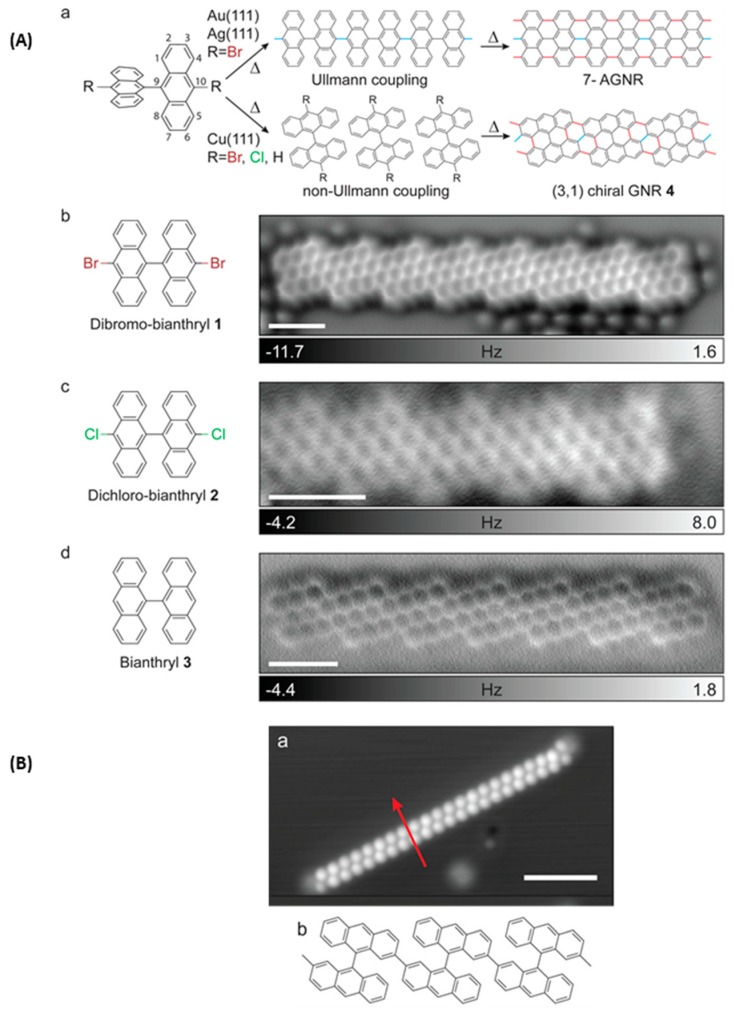
(**A**) GNRs obtained with different bianthracene (BA) precursors. (**B**) Connection of bianthracene precursors at the C2 and C2’ positions (scale bar: 1 nm for all noncontact AFM images). Adapted with permission from Schulz et al. [121]. Copyright 2017 American Chemical Society.

Choice of the surface as well as a careful selection of the shape of the molecular tectons (planar or nonplanar) can impact the geometry of the final GNRs and greatly help in designing GNRs with precise structures [121]. These opportunities were demonstrated with an extensive study carried out on two molecular building blocks, i.e., 10,10′-dibromo-9,9′-bianthracene (DBBA). The debate concerning the molecular arrangement of DBBA on Cu(111) is a long-standing issue [105,107,108,122,123] and by using noncontact atomic force microscopy (nc-AFM), the controversy could be definitely solved. In the specific case of DBBA and despites the presence of bromine atoms, the Ullmann reaction proved to be ineffective on Cu(111) substrates to couple the molecular tectons and GNRs resulting from another coupling mode could be detected on the surface. Interestingly, the same outcome than the one obtained with DBBA could be produced with the unsubstituted 9,9′-bianthracene (BA) or 10,10′-dichloro-9,9′-bianthracene on Cu(111) substrates (see Figure 21Aa–Ad).

Conversely, upon deposition of DBBA on Ag(111) or Au(111), the conventional Ullmann coupling reaction occurred, providing GNRs with armchair edges. By combining STM and nc-AFM at low temperature, the mechanism could be elucidated. Authors demonstrated that upon cleavage of the carbon–halogen bond, the biradicals of DBBA were stabilized by the strong interactions existing with the Cu(111) surface and/or adatoms, so that a severe reduction of the energy barrier for the carbon–hydrogen bond scission at the C2 and C2’ positions could be obtained. Parallel to this, the twisted structures of the different BA derivatives enforced a specific arrangement of the molecular tectons on the surface, favorable to intermolecular interactions at the C2/C2’ positions (see Figure 21(Ba,Bb). As a result of this, and irrespective of the substitution pattern of BA, the same GNRs could be obtained with all monomers, the homocoupling being governed by the C–H scission and not by the recombination of radicals.

### 3.2. Sequential Growth Based on the Coordination Polymer/Glaser Coupling Combination

**Figure 22:**
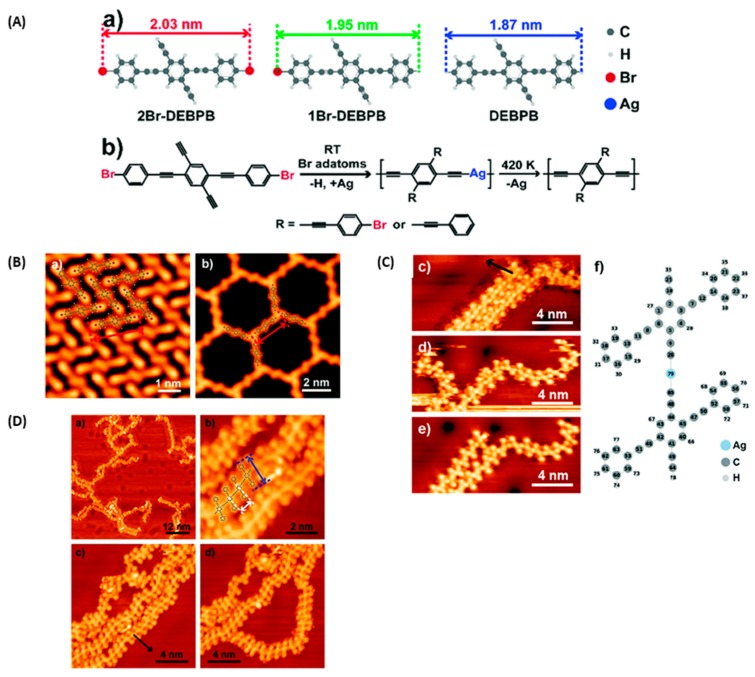
(**A**) Polymerization of 2,5-diethynyl-1,4-*bis*(4-bromophenyl-ethynyl)benzene (2Br-DEBPB) on Ag(111) substrates and chemical structures of 1-((4-bromophenyl)ethynyl)-2,5-diethynyl-4-(phenylethynyl)benzene (1Br-DEBPB) and ((2,5-diethynyl-1,4-phenylene)bis(ethyne-2,1-diyl))dibenzene (DEBPB). (**B**) STM images of the supramolecular phases obtained by annealing the surface at RT temperature for 30 min. (**C**) STM images of the coordination polymer obtained by annealing the surface at RT temperature for 10.5 hours. 4) Covalent polymer chains obtained by annealing the Ag(111) surface at 410K. Reproduced from [124] with permission from The Royal Society of Chemistry.

Still implying intermediate organometallic structures in multistep reactions, a surprising example has been reported in 2018 where the monomer, i.e., 2,5-diethynyl-1,4-*bis*(4-bromophenyl-ethynyl)benzene (2Br-DEBPB) could theoretically react according to the Ullmann and Glaser coupling mechanisms (see Figure 22A) [124]. 

However, only one type of coupling was observed on Ag(111) surfaces. To get this discrimination between the two possible types of coupling, the experiments were carried out at low temperature. Thus, coexistence of two supramolecular phases consisting in a chevron-type structure and a honeycomb arrangement could be found on a Ag(111) surface after a thermal evaporation of 2Br-DEBPB on a surface held at −123 °C (see Figure 22B). After annealing this surface for 1.5 h at room temperature and subsequent cooling of the surface at low temperature (see Figure 22C), a newly ordered structure could be found on Ag (111) surfaces, consisting of parallel rows of molecules where intact molecules and monobrominated 1-((4-bromophenyl)ethynyl)-2,5-diethynyl-4-(phenylethynyl)benzene (1Br-DEBPB) (2% of the total molecules) were coassembled in a densely packed structure. Presence of a large number of bromine adatoms (higher than the 2% resulting from the debromination reaction) on the surface was assigned to the desorption of the fully debrominated molecules. This fact was confirmed by annealing the supramolecular phase for 30 min instead of 1.5 h at room temperature. Twenty-one percent of fully reduced molecules could be found on the surface. However, it has to be noticed that the temperature was insufficient to initiate an Ullmann coupling. Molecular chains corresponding to the formation of coordination polymers could only be obtained by annealing the surface for 10 hours at RT. In these conditions, an activation of the C–H bonds of terminal alkynes could be obtained, resulting in the formation of polymer chains. Finally, conversion of the coordination polymers to covalently bonded polymers was achieved at the relatively low temperature of 137 °C (See Figure 22D). By the abundant presence of bromine adatoms on the Ag(111) surface, interactions between the electron-rich bromines and the electron-deficient hydrogens of terminal alkynes could occur, reducing the BDE, and weakening the C–H bond. As a result of this, Glaser coupling, assisted by both the surface and the presence of halogen adatoms, could occur.

### 3.3. Sequential Growth Based on the Glaser Coupling/Dehydrogenative Coupling Combination

**Figure 23:**
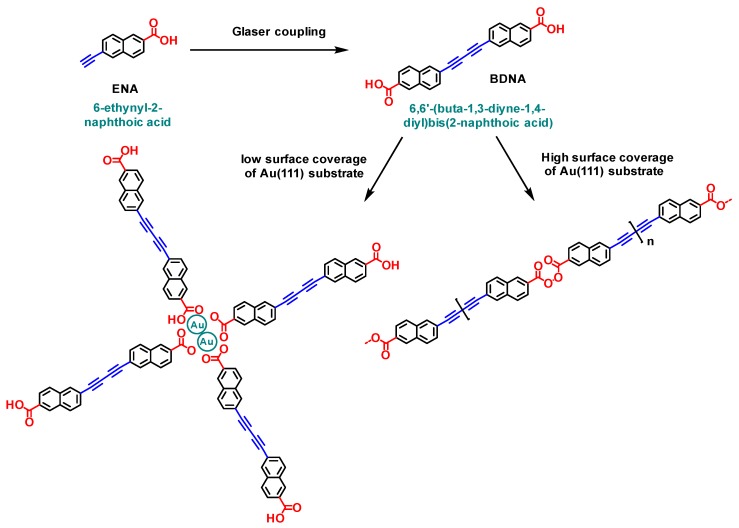
Polymerization of 6-ethynyl-2-naphthoic acid (ENA) by a domino reaction based on the Glaser coupling/dehydrogenative coupling combination. Depending of the density of molecules onto the Au(111) substrates, different structures are obtained.

**Figure 24:**
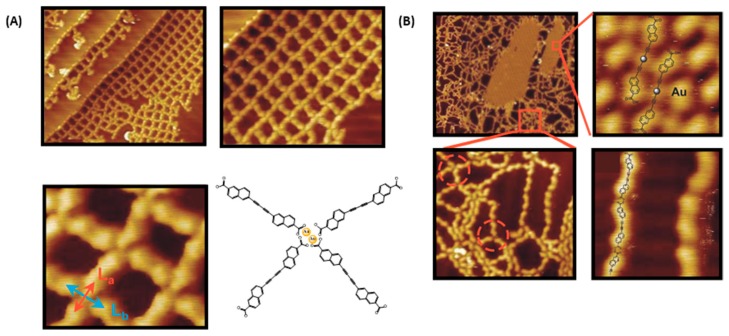
Polymerization of 6-ethynyl-2-naphthoic acid (ENA) by a domino reaction based on the Glaser coupling/dehydrogenative coupling combination. (**A**) STM topographs obtained at low density of molecules after Glaser coupling and formation of the Au-carboxylate complex on Au(111) substrates (left: 42 nm × 38 nm, right: 21 nm × 17 nm, bottom: 5.9 nm × 5.0 nm). (**B**) STM topographs obtained at high density of molecules on Au(111) substrates (top left: 72 nm × 88 nm, top right: 2.6 nm × 1.2 nm, bottom left: 17 nm × 12 nm, bottom right: 5 nm × 4 nm). Reprinted with permission from Held et al. [125]. Copyright © 2016 John Wiley & Sons, Inc.

Convincing results concerning the benefits of a sequential procedure were also obtained by combining a Glaser coupling with a dehydrogenative polymerization [125]. Use of carboxylic acid derivatives for on-surface syntheses of supramolecular networks is not new and this is notably due to the ability of the carboxylic functional groups to form hydrogen bonds or to interact with the metal substrates, giving rise to metal–ligand coordination networks [126,127]. The carboxylic functional groups can also be deprotonated by the metal surface, drastically modifying the structure of the final network. A clear evidence of this influence was provided with trimesic acid deposited onto a Cu(100) surface [128]. Deprotonation of the carboxylic functions modified the adsorption geometry of the network by inducing a rotation of 90° relative to the substrate so that the deprotonated form of trimesic acid stands upright and perpendicular to the surface. With aim at generating covalent bonds, only few articles have been reported in the literature and the decarboxylation of 2,6-naphthalenedicarboxylic acid to form carbon–carbon bonds can be cited as the unique example [129]. Parallel to this, on-surface Glaser coupling of terminal alkynes was reported prior to this work but examples in the literature are scarce [42]. Orthogonality of these two reactions is clear so that their combination was examined for the polymerization of 6-ethynyl-2-naphthoic acid (ENA). Interestingly, influence of the surface topography as well as the density of molecules on surface were determined as controlling the reaction outcome. Upon deposition of ENA on Au(111) substrates at low surface coverage and upon annealing of the surface at 124 °C, a 2D network formed, resulting from the dimerization of ENA, i.e., 6,6′-(buta-1,3-diyne-1,4-diyl)*bis*(2-naphthoic acid) (DBNA) and the formation of a metal–carboxylate coordination network (see Figure 23). Differing from this first arrangement, the same experiments carried out at high surface coverage resulted in the coexistence of two distinct phases on the surface (See Figure 24A). The first one corresponds to an intermediate state of the Glaser dimerization where two ENA molecules are arranged in a linear fashion with Au adatoms ensuring the connection with two ENA units. In the second phase and contrarily to what was expected, the product resulting from the Glaser coupling, i.e., DBNA was detected on the surface, but coexisting with an unexpected compound resulting from an oxidative dehydrogenation. In fact, disordered polymer chains could be detected in this second phase, resulting from the formation of bisacylperoxides (see Figure 24B).

Interestingly, the starting point of these different polymer chains was still a gold–carboxylate complex. As a result of this, the direction of the chains growth is thus predetermined by the geometry of the Au-complex and the number of “ligands” around the Au center. To verify the role of the substrate in the growth direction, similar experiments were carried out onto Au(100) substrates with a structured surface and these latter revealed the Glaser coupling as well as the dehydrogenative coupling to produce chains aligned along with the channels direction, without the formation of ramifications. Polymers extending over 100 nm could be thus prepared, with a thermal stability higher than 160 °C. In this work, the crucial role of the first step, i.e., the formation of the butadiyne groups, was determined as being primordial to activate the formation of bisacylperoxides. Indeed, despite numerous aromatic acids having been examined for on-surface synthesis prior to this work, formation of peroxides from carboxylic groups has never been reported. Finally, XPS experiments confirmed the formation of both Au–carboxylate complexes as well as bisacylperoxide groups.

## 4. Conclusions

In this review, a series of eight combinations of orthogonal reactions enabling the successful realization of 1D and 2D macromolecular organic structures on surface have been reported. The development of efficient chemistries in a vacuum environment is an important challenge conditioning the future of numerous applications. For these reasons, it is of high importance to continue to acquire knowledge and know-how on the growth of macromolecular organic structures in vacuum environment. Based on the different works devoted to the hierarchical or sequential construction of 1D and 2D structures, the possibility to elaborate extended covalent organic structures on surface was brilliantly demonstrated with STM images of scan area sizes larger than 60 × 60 nm^2^. Comparison between the macromolecular structures obtained by sequential or hierarchical growth on surface and those obtained by the classical one-step approach would be of crucial interest for the future development of this approach and the optimization of the reaction conditions. However, the comparison between the two approaches remains extremely difficult with regards to the multiparametric character (metal, crystallographic plane, formation or not of an intermediate supramolecular phase, density of molecules on-surface, etc.) of the delicate growth. This is notably the reason why the comparison between the two models is not established in the different studies. With regards to the large number of reactions existing in organic chemistry and the infinite of combinations, there is still room for improvement and numerous unexplored combinations will be reported in the future.
